# A Case of Procidentia Uteri

**Published:** 1860-11

**Authors:** Gustavus G. Roy

**Affiliations:** Essex County, Virginia


Art. IV.—*A Case of Procidentia Uteri, followed by violent Metritis and Peritonitis; Recovery.* By Gustavus G. Roy, M.D., of Essex County, Virginia.

On the morning of the 29th of April, 1860, I was called very hurriedly to see a negro woman in labor with her fourth or fifth child. On my arrival I met at the door the midwife, who had been with the patient during the night, when she addressed me, as well as I remember, in the following words: "Doctor, we've got a very ugly case; the womb is entirely out, lying on the bed between the patient's legs." I thought if such be the truth we have indeed rather an "ugly case," but I hastened to the bedside of the patient, and, upon examination, found the statement of the old woman very nearly correct.

The patient's condition I found to be one of those rare cases of procidentia uteri so graphically portrayed by Prof. Charles D. Meigs, in his Treatise on Obstetrics, page 101, a representation of a case of which he gives in fig. 40 of the same page. The vagina was as completely inverted as I ever saw the finger of a glove, and the os uteri, grasping the head of the lifeless fœtus, was plainly visible to the eye. At least two-thirds of the gravid womb was projecting, forming a very unsightly tumor, the epithelium of which, from the contact of irritating air, and too frequent handling of the mass by the midwife, had become very dry and the organ very sensitive. The pains were sufficiently good to have expelled the contents of the womb under ordinary circumstances, but the bearing-down efforts of the patient could avail but little to a womb without the axis of the pelvis and entirely beyond their reach. This was sufficiently clear from the fact that whenever I would replace the tumor the very next pain or effort would return it to its unnatural position.

The child being dead, and there seeming to be an utter impossibility of its being delivered by the unaided efforts of the womb, I endeavored to extricate its compressed and deformed head from the grasping fibres of the cervix uteri, which alone seemed to hold it.

With some difficulty I introduced the index finger of each hand between the lips of the os and the sides of the child's head just above its ears, and, steadying the mass with the three remaining fingers and thumb, by compressing the bones of the head, and at the same time making lateral motion, I succeeded in releasing the head, after which the labor was a quick and easy one. A few minutes after the delivery of the fœtus there was a slight contraction of the womb, which brought away the placenta, and with it a large flow of blood. I immediately introduced my right hand into the womb, supporting the mass with my left, returned it and held it in situ until it contracted sufficiently to put a stop to the hemorrhage. After this there was no unnatural or unmanageable flooding, but the "after-pains" were troublesome, and had to be quieted to prevent their again inverting the vagina.

Having directed a little nourishment, and most strenuously enjoined rest and quietude, I left the patient, with the request to be immediately informed should any untoward symptom present itself. Three days afterwards, I was summoned to visit her, when I found her in a most critical condition.

The following symptoms presented themselves: acute pain upon pressure over every part of the abdomen; very great tympanites; strong disposition to diarrhœa; nausea; troublesome hiccough; tongue coated, dry and cracked, and very red around the edges; pulse quick and corded; countenance desponding; lower extremities drawn up, with great pain when extended; kidneys and bladder deficiently performing their duties.

*Treatment.*—Venesection was resorted to until a decided impression was made upon the heart's action, then a large blister was placed over the entire tender surface of the abdomen, which, in order to keep up the vesication, it was necessary to reapply several times. Warm hop poultices were kept constantly over the blistered surface. This plan was rigidly enforced. Internally.—One-fourth of a grain of calomel and one grain of opium were administered every hour, until the bowels were quieted, pain relieved, and rest secured; and a mixture of sweet spirits of nitre, spirits of turpentine, and effervescing citrate of potassa, was directed every four hours. The opium had the desired effect of locking up the bowels, which were permitted to remain unmoved for three days; then a moderate dose of castor-oil was given, which it was necessary to assist with enemata. Several good evacuations were secured, which greatly diminished the tympanites; but pain and tenderness over the abdomen were still very great. Calomel and opium were again resorted to in the same proportion for the same purpose, and with the same result. On the third day several healthy evacuations were secured, with great diminution of the tympanites, pain, and tenderness. After this the calomel and opium were only given as indications required. The stomach now being quite retentive, a mixture of spirits of turpentine, muriate of ammonia, and infusion of serpentaria was directed every four hours. This prescription, together with the blisters and fomentations, was continued until the redness of the tongue, dryness of the skin, fever, and abdominal tenderness disappeared. Mild astringent injections were used to check the unhealthy discharges from the womb, and the patient put upon a course of tonics, under which she slowly improved. I have said nothing of the use of the pessary, because the violence of the inflammation precluded it; in fact, it was unnecessary, as the patient could not, had she the inclination, leave the supine position. As is natural to suppose, it is now necessary for her to use the pessary to keep the womb in situ, for which purpose the gutta-percha ring has been suggested.

